# Editorial: Promoting teamwork in healthcare

**DOI:** 10.3389/fpsyg.2024.1422543

**Published:** 2024-06-14

**Authors:** Juliane E. Kämmer, Margarete Boos, Julia C. Seelandt

**Affiliations:** ^1^Department of Emergency Medicine, University of Bern, Bern, Switzerland; ^2^Institute for Psychology, Georg August University Göttingen, Göttingen, Germany; ^3^Simulation Center, University Hospital Zurich, Zürich, Switzerland

**Keywords:** teamwork, healthcare (MeSH), interprofessional collaboration, Interprofessional education (IPE), patient safety, team performance analysis, Interprofessional communication

## 1 Introduction

Delivering healthcare is inherently collaborative, involving diverse teams across various stages of patient care, from *ad-hoc* emergency and anesthesia teams delivering immediate care to surgeries and tumor boards conferring on long-term cancer treatment (Lamb et al., 2011; Tschan et al., 2015; Choi et al., 2023). Thereby, quality of patient care hinges on the successful intra- and interprofessional collaboration among healthcare professionals, and sensitive interaction with patients and their families (World Health Organization, 2010; Committee on Diagnostic Error in Health Care et al., 2015; Graber et al., 2017). In particular, communication and coordination in healthcare teams are pivotal for team performance and patient safety (Tucker and Edmondson, 2003; Lingard, 2004; Salas et al., 2008; Manser, 2009; Künzle et al., 2010; Fernandez Castelao et al., 2013; Kolbe and Grande, 2013; Tschan et al., 2014). However, achieving effective teamwork is challenging, especially in large hospitals where turnover rates are high, and for interdisciplinary and interprofessional *ad-hoc* teams lacking ongoing collaboration experience (Pearce et al., 2006; Nemeth, 2008; St. Pierre et al., 2011; Fortune et al., 2012). Moreover, healthcare teams face intricate tasks, requiring rapid decision making amidst uncertainty and adaptability to evolving conditions (King et al., 2008; Nemeth, 2008; Fortune et al., 2012). Fostering research into promoting effective teamwork in healthcare stands to significantly enhance patient care quality.

To promote effective teamwork in healthcare, a number of important knowledge and practice gaps need to be closed. The 23 articles in this Research Topic contribute to advancing our understanding of determinants and mechanisms of effective teamwork in healthcare, identifying useful methods for studying teams, and enlarging our repertoire of best practices for promoting and training teamwork in healthcare (see Tables 1, 2). These articles are authored by researchers from countries including Germany, Israel, Sweden, Switzerland, the UK, China, and the USA.

**Table 1:** Overview of articles reporting primarily empirical studies.

**Authors**	**3 keywords**	**Input variables**	**Process variables**	**Output variables**	**Study participants**	**Study design**	**Data type**	**Purpose**	**Results**
Anselmann et al.	Team learning, knowledge sharing, psychological empowerment	Team member characteristics, team characteristics	Individual learning activities, team learning activities	Team effectiveness	Gerontological nurses in teams	Survey study	Questionnaire data	Investigated how individual learning activities influence knowledge sharing and nursing team effectiveness.	Individual learning activities enhance knowledge sharing, leading to improved team effectiveness.
Bienefeld et al.	Human-AI teams, transactive memory, speaking up	Human vs. AI team member knowledge	Interaction with human vs. AI agents	Transactive memory, speaking up, team performance	Intensive care (ICU) human-AI-teams	Field study during simulation training	Observational and performance data	Examined the impact of transactive memory and speaking up in human-AI teams in simulated clinical scenarios.	Interaction with AI positively affects novel hypothesis generation and speaking up, but only in higher-performing teams. Conversely, reliance on human team members negatively affects novel hypothesis generation and speaking up, regardless of team performance.
Dietl et al.	Interprofessional intervention, psychological safety, interpersonal communication	Communication training, perceived psychological safety	Interpersonal communication	Perceived psychological safety, perceived team performance, perceived patient safety risks	Interprofessional teams from obstetric units	Intervention study	Questionnaire data	Examined the psychological mechanisms of a 4-hour communication intervention for healthcare teams aimed at enhancing patient safety and team performance perception by fostering psychologically safe environments and improving communication.	Perceived patient safety risks post-intervention were significantly decreased, whereas no significant changes in interpersonal communication or team performance perception were shown. Mediation analyses revealed interpersonal communication as a mediator between psychological safety and safety performances.
Gerbeth and Mulder	Work engagement, team learning behaviors, dealing with emotions	Amount of work, work pace, cognitive demands, emotional demands	Team learning behaviors, dealing with emotions in the team	Team members' work engagement	Members of interdisciplinary health and social care organizations	Survey study	Questionnaire data	Investigated how team behaviors, such as reflective activities, mediate the impact of work demands on engagement, considering cognitive and emotional dimensions.	Positive associations between work engagement, team learning behaviors, and dealing with emotions in the team were shown. Cognitive demands positively and emotional demands negatively influence work engagement, with team behaviors mediating these relationships.
Kämmer et al.	Teamwork quality, medical teams, team-based diagnosis	Different patient, physician and context factors		Perceived teamwork quality	Emergency physicians	Field study	Questionnaire data	Examined factors affecting perceived teamwork quality in a medical diagnosis setting, where a senior and junior physician team collaborate to diagnose a patient.	Patient case clarity and urgency positively affect perceived teamwork quality, while the level of experience the supervisor has negatively affects both supervisor and trainee perceptions, though to varying extents.
Kolbe et al.	Simulation, education, TeamSIM	Simulation training		Psychological safety, headline reflections, teamwork skills, reaction to TeamSIM	Third-year medical students	Intervention study	Observational and survey data	Developed and evaluated the feasibility of TeamSIM, a simulation-based teamwork training for medical students.	Positive student reactions and increased psychological safety were shown. Students' reflections highlight the effectiveness of the course content, and faculty members rated students' teamwork skills higher after the last compared to the first debriefing.
Körner et al.	Patient safety, error management, training	Blended learning vs. eLearning		Safety-related behaviors in the fields of teamwork, error management, patient involvement, and subjectively perceived patient safety	Interprofessional teams (mainly nurses and physicians) of different wards	Intervention study	Survey data and interview data	Introduced an Interprofessional Training Program (IPTP) employing eLearning and blended learning to enhance patient safety through innovative adult learning methods.	No consistent differences between groups or a clear pattern in safety-related behaviors in the fields of teamwork, error management, patient involvement, and subjectively perceived patient safety were found. Feasibility checks indicate barriers to eLearning participation but highlight increased awareness of patient safety with in-person training.
Schilling et al.	COVID-19, inter-professional teams, mental health	Social support, group identity, professional skills	Personnel's ability to work together and cope with pandemic stress	Delivery of care and staff wellbeing	Health care workers from ICU and those deployed to ICU/COVID wards	Field study	Interview data	Explored the COVID pandemic's impact on teamwork, social dynamics, and mental health among permanent and deployed healthcare workers.	The significance of social factors in teamwork and mental wellbeing, with deployed staff facing increased workload and diminished social support is revealed.
Sheffer Hilel et al.	Professional stereotypes, faultlines, leadership style	Professional stereotypes, team's faultlines	Leadership style	Team's quality of care	Interprofessional teams from geriatric long-term-care facilities	Survey study	Questionnaire data, EHR data on performance	Investigated the impact of professional stereotypes and leadership style on interprofessional team performance and care quality in geriatric long-term-care facilities.	Faultlines are not directly harmful but influence care quality when professional stereotypes emerge. High stereotype teams benefit from person-oriented championship leadership, while low stereotype teams are harmed by it.
Soukup et al.	Cancer multidisciplinary teams, multidisciplinary tumor boards, teamwork among the medical professions		Initiation and interactivity of interaction sequences		Members of MDT meetings in cancer care	Field study	Observational data	Examined MDT meeting dynamics in hospitals.	High interactivity with increased verbal dysfluencies in the latter half of MDT meetings was identified. Findings stress teamwork's critical role in meeting planning, addressing cognitive load, hierarchy, and integration of patient perspectives.
Timm-Holzer et al.	Teamwork in surgery, surgical checklist, intraoperative briefing		Timeout quality		Surgical teams	Intervention study	Observational data	Evaluated team timeout (TTO) quality pre and post StOP?-protocol implementation.	Post-intervention, team timeouts demonstrated higher completeness and engagement, better social atmosphere, and reduced noise, and were less rushed. Contrary to concerns, StOP?-protocols enhance TTO quality without inducing checklist fatigue, highlighting their positive impact on surgical team communication.
Wang et al.	Team functioning, multidisciplinary team, county-level hospitals		Retaining talent, task design, leadership	Team functioning	Hospital presidents, health care team leaders	Field study	Interview data	Examined critical factors shaping team performance from the perspective of leaders in healthcare organizations.	Factors comprise being “stuck in the middle”, local dynamics, talent recruitment hurdles, task focus, and leadership styles. Interventions target talent retention, restructuring of teams, and enhancing collaboration through training.
Weiss M. et al.	Voice/speaking up, psychological safety, team perception	Psycholgical safety	Voice/speaking up	Evaluation of voice as helpful vs. not	Emergency medicine nurses and physicians	Experimental study	Questionnaire data	Examined the impact of nurses voicing work-related concerns on team perception, considering the role of psychological safety.	When psychological safety is high, nurses' input is valued more for team decision-making compared to situations with lower psychological safety.

EHR, electronic health record; ICU, intensive care unit; MDT, multi-disciplinary team.

**Table 2:** Overview of articles reporting method and concept developments.

**Authors**	**3 keywords**	**Development of method or concept**	**Target readers**	**Purpose**	**Results**
Fernández Castillo	Team science, team communication, team coaching	Concept	Healthcare professionals, educators, trainers, team researchers	Build upon ten observations in healthcare team science, emphasizing communication's significance and addressing challenges like accountability and conflict management.	The authors underscore thriving research in interprofessional collaboration, highlighting its evolving understanding and how it boosts teamwork across practitioners' careers.
Kauff et al.	Medical education, intergroup contact, social identity	Concept	Educators, trainers, curriculum developers	Advocate integrating interprofessional education into health-related study programs to address healthcare complexity.	The perspective article emphasizes competency cultivation and fostering diversity appreciation in interprofessional education.
Lia et al.	Intraoperative teamwork, tone, team dynamics	Concept	Team researchers	Propose “tone” as a key factor for understanding team dynamics, linking it to culture, shared mental models, and psychological safety.	The paper provides insights into intraoperative teamwork by elucidating the interplay among culture, shared mental models, and psychological safety.
Morian et al.	Distributed team, team performance, instrument	Method	Distributed emergency teams, team researchers	Investigate the validity, reliability and applicability of the Team Emergency Assessment Measure (TEAM) in distributed healthcare teams.	Report good reliability and validity of the TEAM in distributed acute-care team settings.
Paquette et al.	Perioperative handoffs, teamwork training, patient safety, care coordination, implementation challenges	Method	Educators, trainers	Highlight the risks associated with perioperative handoffs, stressing the importance of teamwork to mitigate miscommunications and ensure patient safety.	Their perspective article underscores the need to address challenges in implementing effective teamwork training programs, emphasizing evidence-based practices.
Schulz and Wirtz	Woman-centered care, interprofessional collaboration, midwifery care	Method	Interprofessional teams, team researchers	Analyzed midwives' perspectives on interprofessional care during pregnancy, birth, and postnatal periods, adapting the Interprofessional Collaboration Scale (ICS).	Report good construct validity in the revised ICS-R.
Seelandt et al.	Research perspectives, team dynamics, interdisciplinary perspectives	Concept	Team researchers	Advocate for diverse perspectives in researching healthcare team dynamics, analyzing a heart surgery team interaction through five lenses.	The paper concludes by suggesting further research avenues and emphasizing the advantages of diverse approaches in healthcare analysis.
Weiss K. E. et al.	Eye tracking, pose estimation, feedback	Method	Medical simulation trainers, team researchers	Utilized minimally invasive video-based technologies like eye tracking and pose estimation to measure teamwork in healthcare simulation training with medical students.	The authors emphasize the potential of these objective metrics in creating visualizations of team interactions, stressing the need for further research.
Wespi et al.	Objective process measures, medical simulation training, team performance assessment	Concept, method	Medical simulation trainers, team researchers	Explore the potential of the use of objective measures for evaluating team performance for research and training purposes by conducting expert interviews.	Identify various potential objective measures including acoustical, visual, physiological, and endocrinological measures. Highlight the opportunities and challenges (e.g., feasibility, complexity, cost, and privacy concerns) of assessing healthcare team performance using objective process measures.
Witti et al.	Conceptual framework, healthcare education, interprofessional collaboration	Concept	Team researchers, educators, trainers, curriculum developers	Present FINCA, a conceptual framework aimed at improving interprofessional collaboration in healthcare education and practice with key variables and activities essential for problem-solving competency among healthcare professionals.	The framework aims to support curriculum development, assessment of outcomes, and foster interprofessional problem-solving skills across healthcare settings.

## 2 Overview on the articles in this Research Topic

One way of grouping the articles relates to the well-known and widespread input-process-outcome model of teamwork (Ilgen et al., 2005; Hackman, 2012), another is to group them along the methodical dimension. Providing a brief overview of variables and topics covered, Table 1 comprises our categorization based on these two taxonomies for the 13 articles that report primary empirical studies. Input variables considered in the studies range from member and team characteristics, diverse professional knowledge, skills and stereotypes, and task-based cognitive and emotional demands, to the experimental induction of communication training, simulation and E-learning. The articles focus on multiple process variables including e.g., learning activities, coordinative behavior, interaction with human and AI agents, speaking up behavior, and coping with stress. Output variables considered include e.g., team effectiveness and team skills, psychological safety, patient safety, as well as team wellbeing. The Research Topic comprises articles based on various kinds of data, ranging from questionnaire and interview data to observational data, and performance measures. Besides medical students, a large range of healthcare professionals participated in the studies, individually and as teams.

Table 2 contains the 10 contributions that focus on new methods and concepts. Topics range from presenting new measures for assessing interprofessional teamwork, to proposing conceptual frameworks aimed at improving interprofessional collaboration and education in healthcare, and advocating for diverse perspectives in researching healthcare team dynamics.

## 3 Discussion

As we move forward, three crucial next steps emerge, each essential for advancing our understanding and practice in this critical area.

Firstly, given the rapidly evolving nature of the healthcare domain, encompassing technological advancements, clinical research, and evolving work environments, research continually faces emerging research questions. To tackle these, leveraging insights from existing research in tandem with innovative methodologies is particularly promising. For example, the utilization of advanced technologies such as eye tracking, as delineated by Weiss K. E. et al., in the examination of human-AI teams (Bienefeld et al.), presents a novel approach to understanding attention dynamics within these teams. Additionally, integrating biophysiological process measures (Wespi et al.) with traditional observer ratings (e.g., Morian et al.) and self-reports (e.g., Kämmer et al., Schulz and Wirtz) offers potential for enriching our understanding of the multifaceted nature of teamwork across various levels. Furthermore, exploring alternative viewpoints such as the temporal or conflict-power-status perspectives, as advocated by Seelandt et al., is likely to yield valuable new insights.

Secondly, research and curriculum development must prioritize the provision of practically relevant insights and methods to prepare practitioners for one of the biggest challenges in healthcare: interprofessional collaboration. The Team FIRST framework identifies 10 essential teamwork competencies for healthcare providers (Greilich et al., 2023) that could guide further research in real-world settings. Another approach toward this goal involves tailoring research designs and samples to reflect the interprofessional reality, for instance by involving diverse members of surgical teams with different backgrounds (e.g., Timm-Holzer et al.). Additionally, conducting more field and observational studies, as demonstrated by Schilling et al.'s field study during the COVID-19 pandemic or Soukup et al.'s investigation of real-life cancer multidisciplinary team meetings, proves essential. It is also imperative to validate findings from the laboratory in practical settings, exploring the boundary conditions of existing findings and methodologies in diverse environments and adopting a condition-focused approach (Hackman, 2012). For instance, Fernández Castillo et al. emphasized that more communication may not invariably lead to improved outcomes; instead, contextual factors influence the value of communication, which need to be scrutinized in further research.

Lastly, ensuring the accessibility of research findings and knowledge for interprofessional education is essential for preparing the next generation of healthcare professionals. While this Research Topic showcases innovative developments in interprofessional education (e.g., Körner et al., Kolbe et al., Witti et al.), the focus should now shift toward increasing the accessibility of educational materials and resources. This could range from publishing open-access materials alongside research articles, as done by Körner et al., to establishing platforms dedicated to sharing interprofessional training materials and curricula (e.g., https://www.did-act.eu, https://did-act.instruct.eu/course/view.php?id=3), such as virtual patient case collections (e.g., https://icovip.eu/) and the initiative Behavioral Science Applied to Healthcare (BSAH; Keller et al., 2024). By making such resources readily available, we can empower healthcare professionals with the necessary skills and knowledge to effectively collaborate across disciplines, ultimately enhancing teamwork and patient outcomes.

By embracing these challenges and opportunities, we can further enhance our understanding and practice of effective collaboration in healthcare settings, ultimately leading to improved patient care quality and outcomes.

## Author contributions

JK: Conceptualization, Writing – original draft, Writing – review & editing. MB: Conceptualization, Writing – original draft, Writing – review & editing. JS: Conceptualization, Writing – original draft, Writing – review & editing.
